# Supplementary material for: Mimicking reductive dehalogenases for efficient electrocatalytic water dechlorination
Source: Nat Commun. 2023 Aug 23;14:5134. doi: 10.1038/s41467-023-40906-6 (PMC10447495; doi:10.1038/s41467-023-40906-6)
Supplement: Supplementary file 1 — Supplementary Information [file 41467_2023_40906_MOESM1_ESM.pdf]

Supplementary Information for

Mimicking reductive dehalogenases for electrocatalytic water dechlorination

Yuan Min[1], Shu-Chuan Mei[1], Xiao-Qiang Pan[1], Jie-Jie Chen[1]*, Han-Qing Yu[1]*, Yujie Xiong[2]*

[1]Department of Environmental Science and Engineering, University of Science and Technology of China, Hefei, Anhui 230026, China
[2]Hefei National Research Center for Physical Sciences at the Microscale, Collaborative Innovative Center of Chemistry for Energy Materials (iChEM), School of Chemistry and Materials Science, University of Science and Technology of China, Hefei, Anhui 230026, China.
*email: chenjiej@ustc.edu.cn; hqyu@ustc.edu.cn; yjxiong@ustc.edu.cn

Contents:

Supplementary Methods

Supplementary Notes 1-5

Supplementary Figures 1 to 36

Supplementary Table 1

Supplementary References (1–23)

1. Supplementary Methods

Chemicals

Dicyandiamide, B_{12} and CoPc were obtained from Aladdin Co., USA. Nafion perfluorinated resin solution (5 wt.%) was obtained from Sigma–Aldrich Co., USA. Single-layer graphene oxide aqueous dispersion solution was purchased from XFNANO Materials Tech Co., China. All the chemicals were analytical grade and used without further purification. Deionized water (DI, R=18.25 MΩ) was used in all experiments.

Synthesis of N-doped graphene

The N-doped graphene nanosheets were prepared with a modified method reported previously[1]. The graphene oxide (5 mg mL^{-1}, 3 mL) and dicyandiamide (84 mg) were dissolved in DI water (27 mL) with stirring. Then, the homogeneous solution was transferred into Teflon-lined stainless steel autoclave (50 mL), heated to 180 °C and kept for 24 h. The reaction mixture was cooled to room temperature, washed repeatedly in water, and dried under vacuum at 60 °C.

MD simulations

To gain theoretical insights into the sandwich-like assembly of molecular catalyst with GO nanosheets, classical MD simulations were performed. A cubic cell of 10.23 × 10.23 × 10.23 nm^3 was constructed to simulate the aqueous solution. The initial

distance between two GO nanosheets (10.23×4.92 nm^2) was 2 nm, which can be optimized by a dynamic balance of the solvation effect and the non-bonded interactions between sheets in the MD simulation. The number of molecular catalysts (B_{12} or CoPc) was 24, which is sufficient for a distribution over the top surface or the interlayer space. The GO nanosheets, B_{12}, CoPc and dichloroacetate ions were all modeled with the GROMOS 54a7 force field[2]. The water molecules were described by the SPC/E model[3]. To probe the intercalation behaviors of ions into the interlayer nanospace, dichloroacetate ions were randomly placed in the solution away from the graphene nanosheets.

The system was first minimized for 50,000 steps (timestep = 2 fs) using the steepest descent method. Temperature (300 K) and pressure (1 atm) were maintained by the Berendsen thermostat and Parrinello–Rahman barostat[4,5]. Production simulations were performed at constant pressure and temperature for 20 ns. Bonds involving hydrogen atoms were constrained using the LINCS method[6]. Short-range electrostatic and van der Waals interactions were calculated by the Lennard–Jones pairwise potential[7]. The potential was switched off smoothly at a cutoff distance of 2 nm. Long-range electrostatic interactions were evaluated using the particle mesh Ewald algorithm with a Fourier spacing of 0.16[8].

All MD simulations were performed using the GROMACS 2018 package, and the trajectories were analyzed with the GROMACS utilities[9]. Movies of the simulation trajectories were generated with the Visual Molecular Dynamics program[10]. Figures of the simulation systems were produced using PyMOL software[11,12].

For each MD simulation, the second 10-ns stage of the molecular dynamics trajectory was used for statistical analysis. The radial distribution function of water oxygen around DCA, $g(r)$, is defined by the following equation (1):

$$g_{DCA-O_w}(r) = \frac{\langle \rho_{O_w}(r) \rangle}{\langle \rho_{O_w} \rangle_{local}} = \frac{1}{\langle \rho_{O_w} \rangle_{local}} \frac{1}{N_{DCA}} \sum_{i \in DCA}^{N_{DCA}} \sum_{j \in O_w}^{N_{O_w}} \frac{\delta(r_{ij} - r)}{4\pi r^2} \qquad (1)$$

where O_W is the O atom of water, $\langle \rho_{O_w}(r) \rangle$ is the particle density of O_W at a distance r around DCA, and $\langle \rho_{O_w} \rangle_{local}$ is the particle density of O_W averaged over all spheres around DCA within half of the box length. N_{DCA} is the number of DCA, and N_{O_w} is the number of O_W.

The coordination number (*CN*) of water molecules around DCA at different positions was calculated by the following equation:

$$CN(r) = 4\pi\rho_N \int r^2 g(r) d(r) \qquad (2)$$

The hydrogen bonds within a distance of 4 Å to DCA were identified by the donor–acceptor pair such as O–H_{wat}···O_{wat} and O–H_{wat}···A, where A is the oxygen of DCA or the nitrogen of B_{12}. The interlayer distance distribution along the simulation time was determined by the minimum distance between the graphene nanosheets. The distance between each CoPc molecule and the graphene nanosheet was obtained using the same method.

DFT calculations

Spin-polarized calculations were carried out with DFT calculations using the DMol^3 code[13]. The electron–electron interactions were described using the generalized gradient approximation (GGA) and the Perdew, Bueke and Ernzerhof (PBE) functional with all electrons included[14,15]. For all structures, the minimum energies were obtained until the energy became less than 1×10^{-5} Ha. The atoms were fully relaxed without symmetry restrictions until the maximum force was less than 0.002 Ha Å^{-1}, and the maximum displacement was within 5×10^{-3} Å. To consider the effect of long-range dispersion interactions, the semiempirical Tkatchenko–Scheffler (TS) scheme was utilized[16]. Since all the systems were in aqueous solution, rather than in vacuum, the water environment was taken into account using the conductor-like screening model (COSMO)[17]. The self-consistent field (SCF) tolerance was set to 1×10^{-6} Ha with a smearing value of 0.005 Ha for the geometrical and electronic calculations.

To model the cobalt-centered molecular catalysts, a planar corrin ring coordinated with cobalt (Co-corrin), cobalt phthalocyanine (CoPc) and an axially 5,6-dimethylbenzimidazole ligated Co-corrin (called the simplified B_{12}) were constructed. Since the pKa values of chloroacetic acids (tri-, di- and mono-) are lower than neutral pH, the substances can be completely ionized to form three chloroacetate ions. For the partial density of states (PDOS) calculation, a large cell of $2.5 \times 2.5 \times 3$ nm^3 was built for simulating the isolated small molecular system with a k-point sampling of $1 \times 1 \times 1$. Based on the MD simulations, the local PDOS analysis was conducted for a solvated dichloroacetate ion with the number of water molecules varying from 0 to 5.

2. Supplementary Notes

Supplementary Note 1

MD simulations were performed using alternative water model (TIP3P) to describe the water confinement. Simultaneously, the run time has been extended. Each system was simulated for 50 ns to investigate the equilibrium state of the layered structures. The structural profile at the inception of the simulation (0 ns) reveals the discernible separation of the GO sheets, along with the initial assembly of B_{12}. This early configuration provides crucial insights into the initial state of our system. During the simulations, GO and B_{12} changed their position and orientation to reduce steric hindrance. It was observed that the inserted B_{12} in the interlayers positively improved the structural stability, which can be attributed to the interactions between B_{12} and GO (Supplementary Fig. 2c). Compared with GO-B_{12} (Supplementary Fig. 2b), GO-B_{12}-GO exhibits a distribution of B_{12} with less over-stacking. Additionally, density profiles (Supplementary Fig. 3) show that the density of water at the solution-electrode interface for GO-B_{12}-GO is lower that compared to GO-B_{12}. The intercalated B_{12} exhibits a less solvated structure than the surface-adsorbed B_{12}. Consequently, these findings emphasize the vital contribution of B_{12} in bolstering structural stability.

Root mean square deviation (RMSD) profiles were used to assess the structural stability and convergence of the system over time. By monitoring the GO sheets of GO-B_{12} and GO-B_{12}-GO electrodes (Supplementary Figs. 4a and b), the RMSD exhibits a value of ~0.1 nm, indicating that the structures converged in 50 ns. To quantify the strength of the interactions between GO sheets, the non-bonded interaction energies

were decomposed to the Coulombic short-range (Coul-SR) energy and Lennard-Jones short-range (LJ-SR) energy. Even though the decomposed energy quantity is not a binding energy, it serves as a useful parameter for monitoring the system throughout the simulation process. The average short-range interaction energies remained relatively constant with minor variations (Supplementary Figs. 4c and d).

Supplementary Note 2

To investigate the structural and chemical compositions across the layers in the sandwich-like configuration of GO-B_{12}-GO, SRPES measurements were employed. The C 1*s* spectra indicate the presence of C=C graphitic components and oxygen-containing groups (Supplementary Fig. 6), such as C=O groups from the side chain (-$CONH_2$) of B_{12} molecules, as well as C–O and O–C=O groups of GO sheets[18]. At a constant level of photon energy, the atomic fractional ratio of carbon in different chemical states was determined by the ratio of peak areas. As shown in Supplementary Fig. 7, the ratio of C=O/C–O is 1.4 in GO-B_{12} and1.0 in GO-B_{12}-GO at a photon energy of 460 eV (at a depth of ~2.14 nm), suggesting the lower loading ratio of B_{12} at the outer surface in GO-B_{12}-GO compared to that of GO-B_{12}. Moreover, the ratio of oxygen-containing C and sp^2 C indicates a larger loading ratio of GO in GO-B_{12}-GO compared to GO-B_{12}. With an increase in photon energy up to 940 eV (at a depth of ~4.15 nm), the C 1*s* spectra show a shift of binding energy, suggesting the dual components of C=O/C–O at surface to the single component of C–O at subsurface. Thus, at the photon energy of 940 eV, a dominant component of GO was found in the layers below surface. Similarly, N 1*s* spectra also confirm the presence of –$CONH_2$ side chain from B_{12} (Supplementary Fig. 8). Therefore, the spatial distribution of chemical components across layers ranging from 2~4 nm is confirmed by SRPES spectra in both GO-B_{12} and GO-B_{12}-GO electrodes.

Supplementary Note 3

Fourier-transform infrared (FTIR) spectra (Supplementary Fig. 16) show distinct peaks[19] for B_{12}, including the cyanide stretching frequency at 2133 cm^{-1}, amide side chains at 1662 cm^{-1}, and breathing modes of the corrin ring at 1575 and 1550 cm^{-1}. In the fingerprint region, the GO-B_{12} electrode exhibits the presence of two additional peaks at 1218 and 1145 cm^{-1} compared to GO. Meanwhile, a noticeable red shift of the band, specifically to 1203 cm^{-1}, is observed in the case of the GO-B_{12}-GO heterostructure.

To understand the contribution of the intercalated B_{12} to the enhanced activity, *in situ* ATR-FTIR studies were employed. In the case of GO electrode (Supplementary Fig. 17a), a significant increase of absorption peak at 1643 cm^{-1} was detected upon absorption of water. This peak is assigned to the C=C skeletal vibrations of graphitic domains or the deformation vibration of intercalated water[20]. A decrease in peak area is observed under the electrochemical conditions with applied potential from −0.2 to −1.2 V *vs*. Ag/AgCl. Specifically, the absorption peaks at 1040 cm^{-1} and 1392 cm^{-1} exhibit a significant decrease at -0.8 V, suggesting the reduction of C–OH groups[21]. Simultaneously, a new peak at 1207 cm^{-1} appears at –1.0 V, indicating the formation of ethers. Furthermore, another new peak appears with increasing intensity at 1566 cm^{-1} from –0.8 V to –1.2 V, which is attributed to the formation of vibrational stretching of C=C, suggesting the formation of graphitic carbon components. It is worth noting that the peak intensity at 1643 cm^{-1} also decreases, which can be ascribed to a loss of intercalated water[20,22].

In the case of GO-B_{12} and GO-B_{12}-GO, the characteristic peaks of B_{12} are overlapped by the strong GO bands. Thus, reduction of GO was employed as a probe reaction to reflect the differences between GO-B_{12} and GO-B_{12}-GO electrodes under electrochemical conditions. The reduction of GO sheets is slower in GO-B_{12} compared to GO alone (Supplementary Fig. 17b), suggesting that the reduction current is diverted to the reduction processes of Co center in B_{12}. The GO-B_{12} electrode exhibits a relatively unchanged peak at 1632 cm^{-1}, indicating the vibrations of C=C domains or the deformation vibration of intercalated water remained. In contrast, the GO-B_{12}-GO shows a fastest reduction of GO sheets (Supplementary Fig. 17c), indicating that the intercalated B_{12} in the GO-B_{12}-GO electrode facilitates the mediation of protons and electrons, leading to the enhanced reduction of GO sheets within the electrode.

Moreover, FTIR absorption peaks of water show a high correlation with the microenvironments within various electrode configurations. From air phase to aqueous phase, the asymmetric wide peaks appearing between 2,750 and 3,750 cm^{-1} can be characterized by five specific hydrogen-bonding (HB) configurations, including H_2O at ~3636 cm^{-1}, which lacks HBs; H_2O at ~3570 cm^{-1} (DDA), which donates two HBs and accepts one; H_2O at ~3430 cm^{-1} (DA), which donates one and accepts one HB; H_2O at ~3220 cm^{-1} (DDAA), which both donates and accepts two HBs; and H_2O at ~3040 cm^{-1} (DAA), which donates one and accepts two HBs[23]. In the case of GO, based on the above analysis of GO reduction and restacking process, Supplementary Fig. 18a shows a loss of H_2O and a formation of $–CH_2$ groups (1850 and 1785 cm^{-1}). This can be attributed to the increased ratios of DA/DDA or DA/DDAA. Similar change of this

peak was found in GO-B_{12}-GO with a more significant trend (Supplementary Fig. 18c). In the case of GO-B_{12}, the edge of this peak at the high wavenumber remains unchanged, suggesting no loss of water in GO-B_{12}, and the $-CH_2$ peaks are relatively weak (Supplementary Fig. 18b). Therefore, the intercalated B_{12} in GO-B_{12}-GO plays a critical role in facilitating the electrochemical reduction.

Supplementary Note 4

To explore the applicability of the sandwich-like electrode (GO-mol-GO, mol: molecular catalyst), cobalt phthalocyanine (CoPc) was used as an alternative cobalt-centered catalyst (Supplementary Fig. 21). The GO-CoPc-GO electrode consisted of GO and CoPc molecules (Supplementary Fig. 22a). XAFS spectra were used to identify the R space for the GO-CoPc-GO electrode. A dominant Co–N coordination bonding was identified, while no peak of Co–Co coordination was found at higher R values (Supplementary Fig. 22b-d). XRD patterns indicate that the GO-CoPc-GO sheets had peaks at 7.16°, 9.36°, 18.38° and 18.78°, corresponding to an interlayer spacing ranging from 1.23 to 0.47 nm (Supplementary Fig. 23). The various interlayer distances are attributed to the π–π stacking of CoPc molecules as illustrated in snapshots of MD simulations (Supplementary Fig. 24a and c). The distribution of the distance between each CoPc and the GO nanosheet displays a more compact stacking of the GO-CoPc than the GO-CoPc-GO (Supplementary Fig. 24b and d). Since the size of the inner nanospace varies with the molecular catalysts, a large variety of Co-centered molecular catalysts would be expected to be available for tuning the reaction space.

Supplementary Note 5

To prepare a similar configuration as the GO-B_{12}-GO, graphene nanosheets were doped with nitrogen to form the GN-B_{12}-GN electrode. The structural information of the GN-B_{12}-GN electrode was examined by high-resolution X-ray photoelectron spectroscopy (XPS) analysis. For the C 1*s* spectra (Supplementary Fig. 30a), the peaks at 285.4 and 287.8 eV suggest the formation of C=N and C−N bonds. For the N 1*s* spectra (Supplementary Fig. 30b), the characteristic peaks at 398.7, 399.8 and 400.4 eV are assignable to pyridinic N, pyrrolic N and graphitic N, respectively. Additionally, the Raman spectra (Supplementary Fig. 31a) display peaks at 1352.8 and 1593.9 cm^{-1}, corresponding to the D band and G band, respectively. The ratio of the two peak (I_G/I_D) intensities can be used to indicate the graphitization of the carbon support. GN shows a lower degree of graphitization than GO, which is consistent with the TEM observations (Supplementary Fig. 31b).

After applying similar electrochemical tests for the GO-B_{12}-GO), the contribution of the GN-B_{12}-GN electrode to TCA reduction is ascribed to the tightly bound B_{12}, while the loosely bound B_{12} does not increase the reduction current density (Supplementary Fig. 32). Therefore, the applicability of the sandwich-like configuration can be extended to other carbon supports.

4. Supplementary Figures and Tables

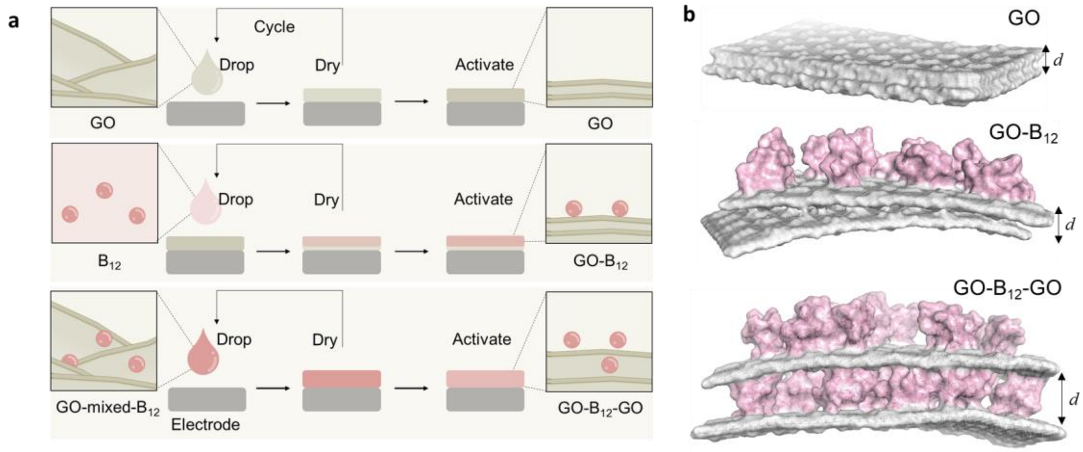

Supplementary Fig. 1 | a Schematic illustration of the preparation of electrodes, with steps involving drop–dry cycles, electrochemical activation and ultrasonication. **b** MD simulations of the as prepared GO, GO-B_{12} and GO-B_{12}-GO electrodes.

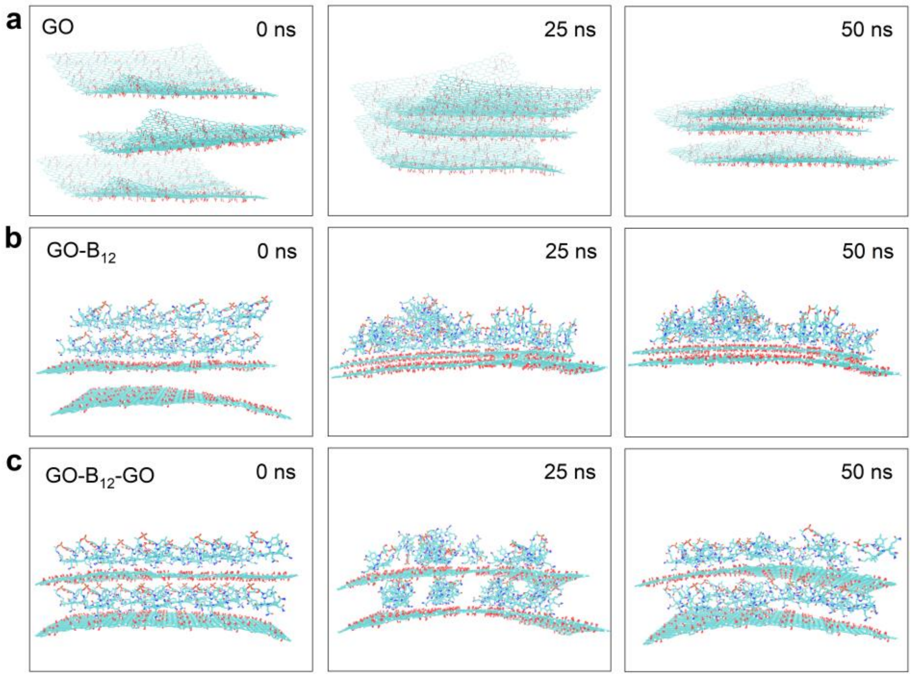

Supplementary Fig. 2 | Snapshots of MD simulations for the layered models of **a-c** GO, GO-B_{12} and GO-B_{12}-GO with water molecules hidden for visualization.

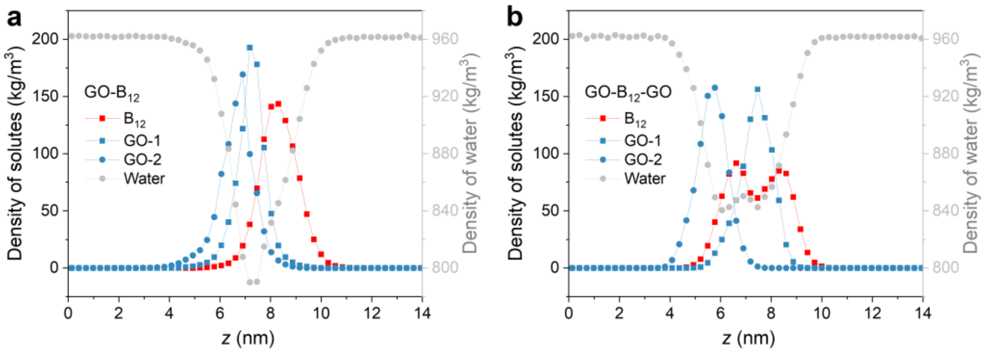

Supplementary Fig. 3 | Density profiles for **a** GO-B_{12} and **b** GO-B_{12}-GO in water solution. GO-B_{12}: upper GO sheet supporting B_{12} molecules (denoted as GO-1), lower GO sheet (denoted as GO-2). GO-B_{12}-GO: upper and lower GO sheet (denoted as GO-1 and GO-2).

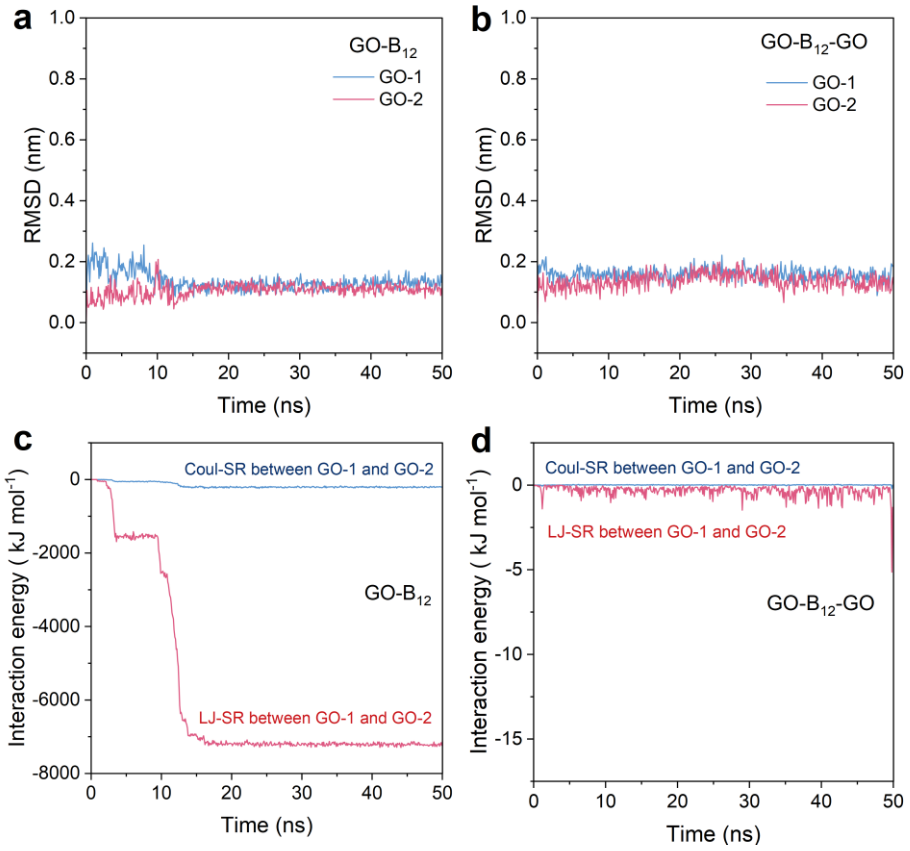

Supplementary Fig. 4 | RMSD curves and the non-bonded interaction energy profiles of GO sheets for **a, c** GO-B_{12} and **b, d** GO-B_{12}-GO electrodes during the simulation.

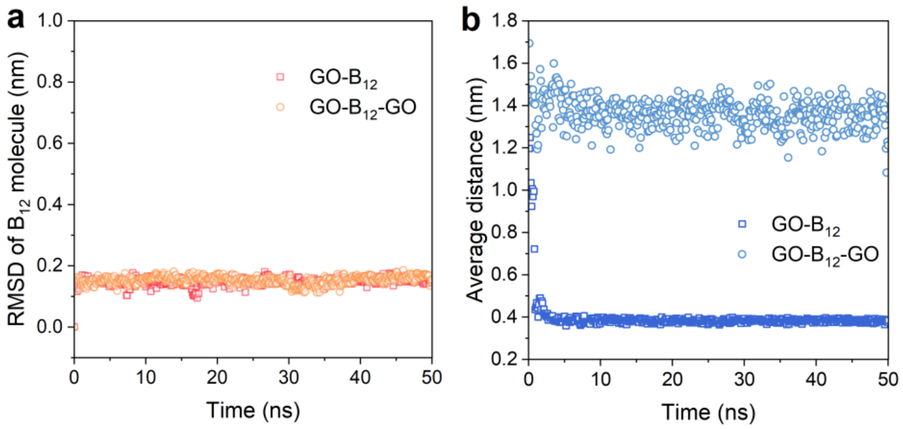

Supplementary Fig. 5 | RMSD curves of B_{12} and interlayer spacing profiles of **a** GO-B_{12} and **b** GO-B_{12}-GO.

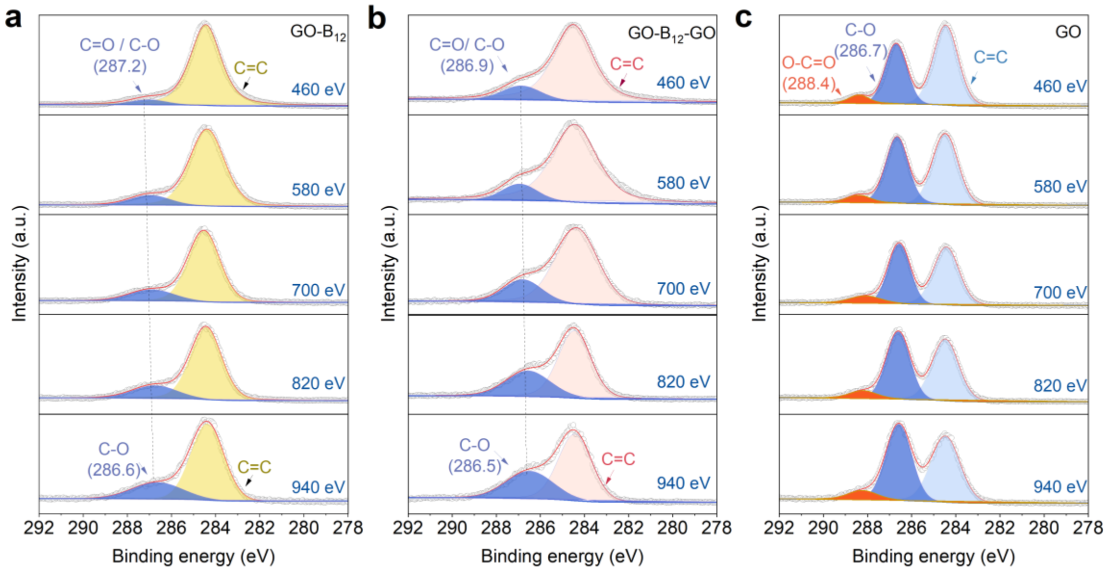

Supplementary Fig. 6 | SRPES spectra of different electrode configurations recorded under the photon X-ray energies of 460, 580, 700, 820 and 940 eV, respectively. C 1*s* spectra obtained by SRPES for **a** GO, **b** GO-B_{12} and **c** GO-B_{12}-GO.

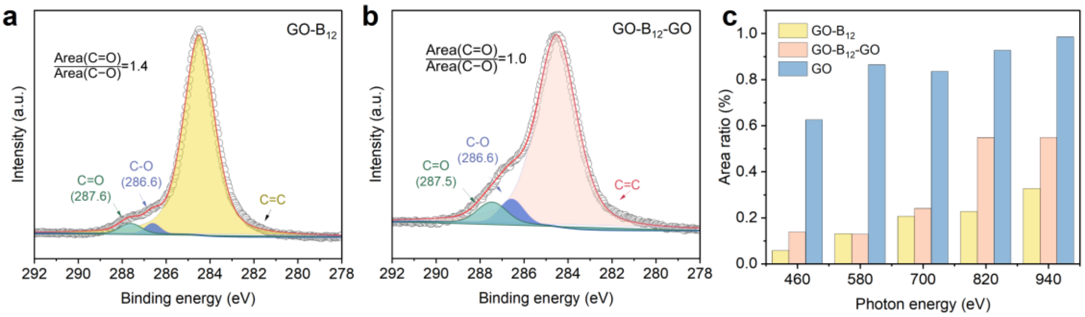

Supplementary Fig. 7 | C 1*s* SRPES spectra and the area ratio of different C states for **a** GO-B_{12} and **b** GO-B_{12}-GO. **c** The area ratio of O states to C states.

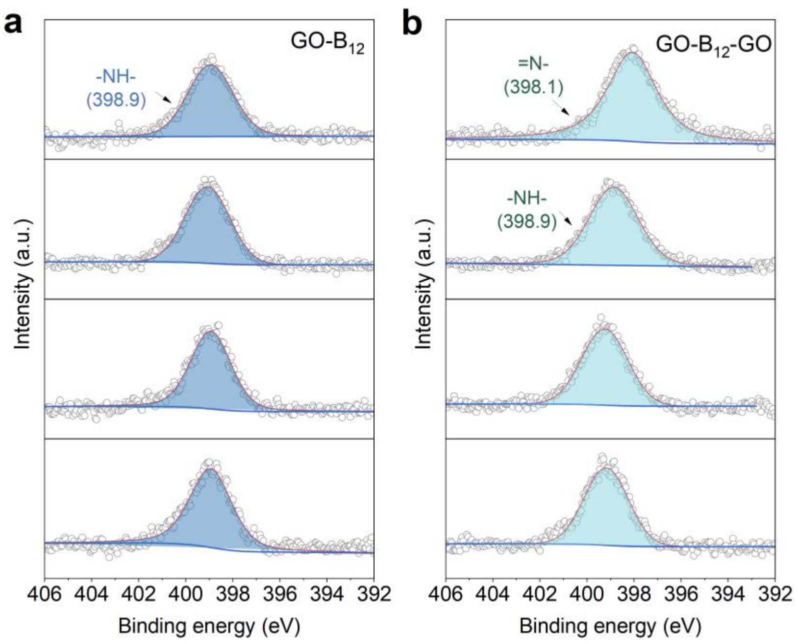

Supplementary Fig. 8 | N 1*s* SRPES spectra of **a** GO-B_{12} and **b** GO-B_{12}-GO.

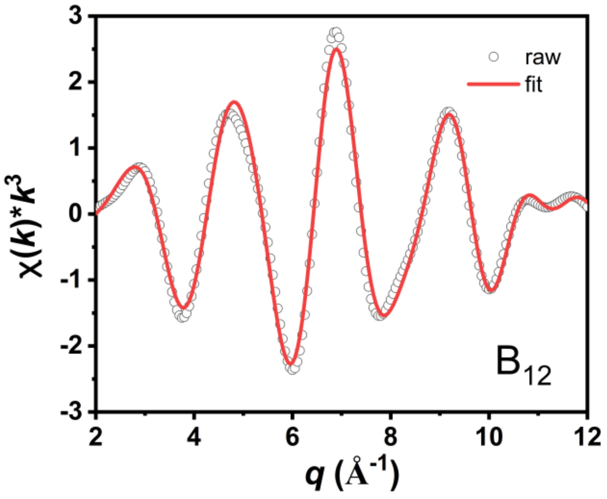

Supplementary Fig. 9 | The corresponding Co K-edge EXAFS fitting in *q* space of B_{12}.

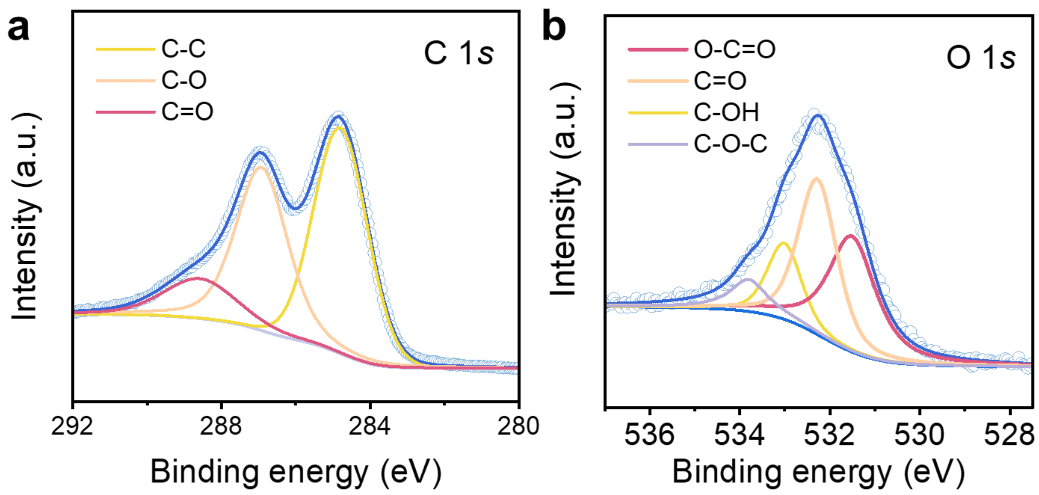

Supplementary Fig. 10 | High-resolution XPS of **a** C 1*s* and **b** O 1*s* for the GO support.

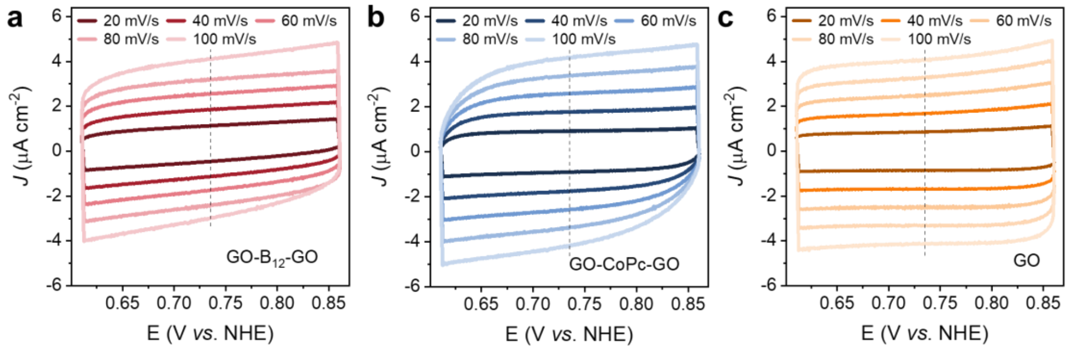

Supplementary Fig. 11 | CV curves at different scan rates for the **a** GO-B_{12}-GO, **b** GO-CoPc-GO and **c** GO electrode, pH=7.0.

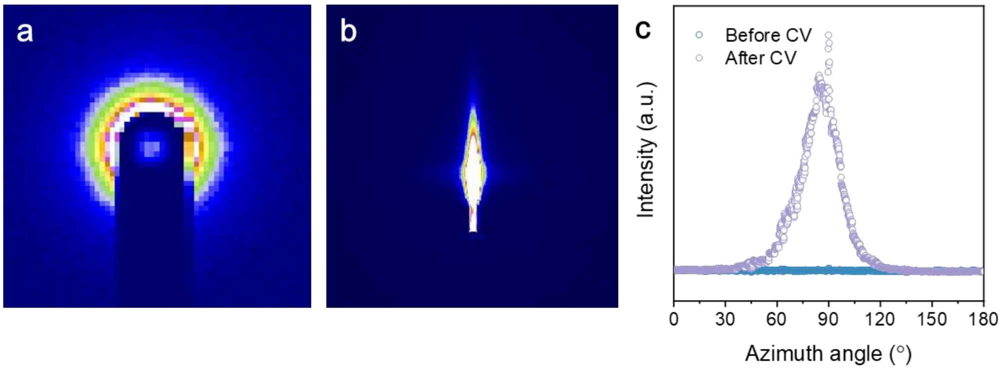

Supplementary Fig. 12 | Small-angle X-ray scattering (SAXS) patterns of GO-B_{12}-GO obtained before (**a**) and after (**b**) electrochemical treatments with (**c**) the azimuthal angle (α) plots. Before electrochemical treatment, the scattering results indicate a random or isotropic distribution of GO components. The well-defined isotropic scattering at $\alpha = 90°$ suggests the enhanced alignment of GO sheets and the formation of a layer-by-layer structure in GO-B_{12}-GO after electrochemical treatment.

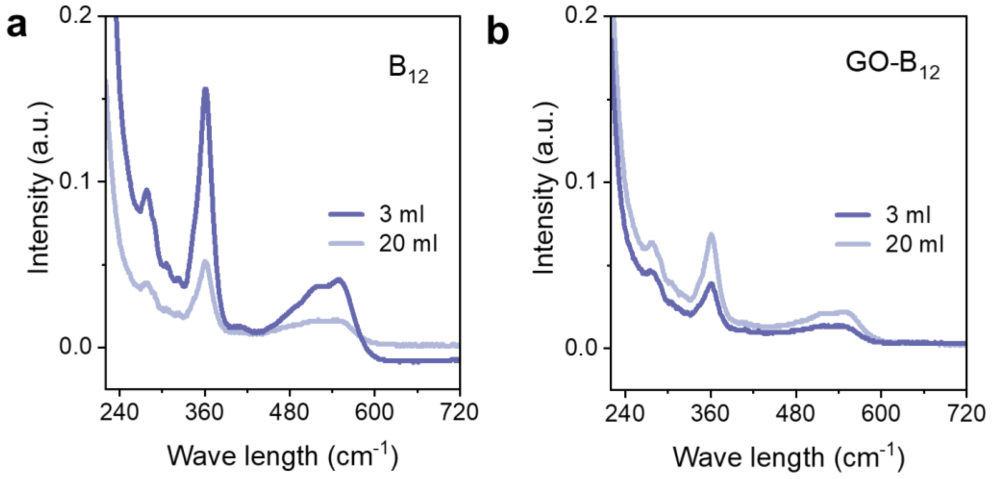

Supplementary Fig. 13 | UV-vis absorption spectra of the collected B_{12} solutions of **a** the B_{12} electrode and **b** the GO-B_{12} electrode.

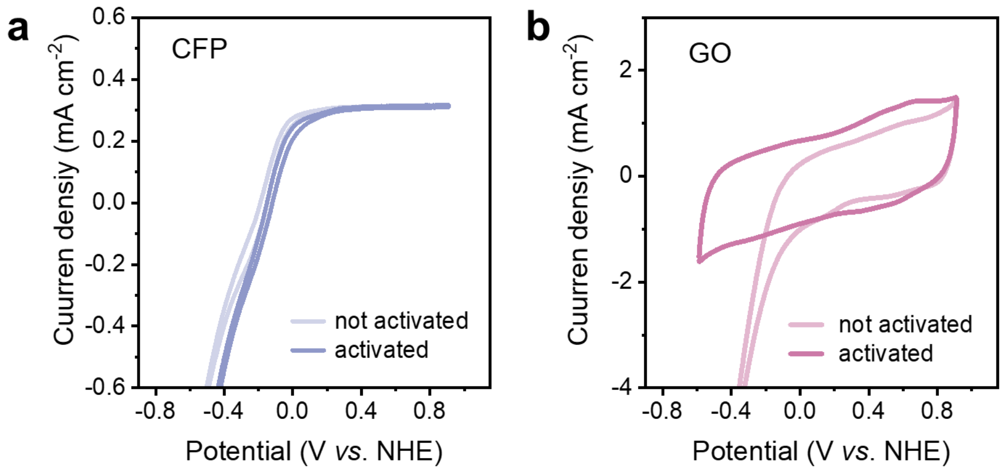

Supplementary Fig. 14 | CV curves of **a** the blank carbon fiber paper electrode (CFP) and **b** the blank GO electrode at a scan rate of 200 mV/s, pH=7.0.

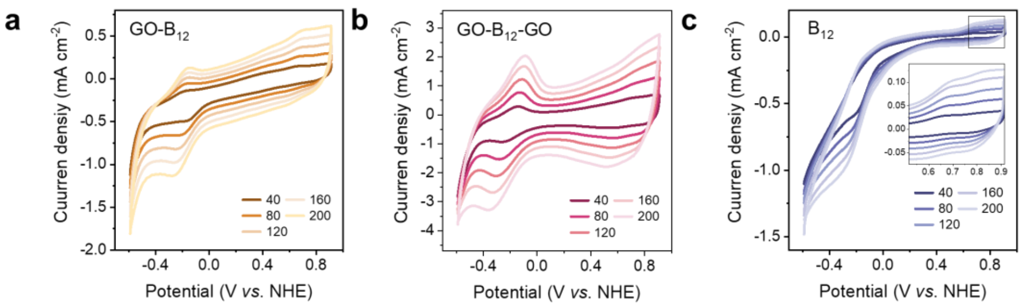

Supplementary Fig. 15 | CV curves of **a** the GO-B_{12} electrode, **b** the GO-B_{12}-GO electrode and **c** the B_{12} electrode without GO support at different scan rates (40, 80, 120, 160 and 200 mV/s), pH=7.0.

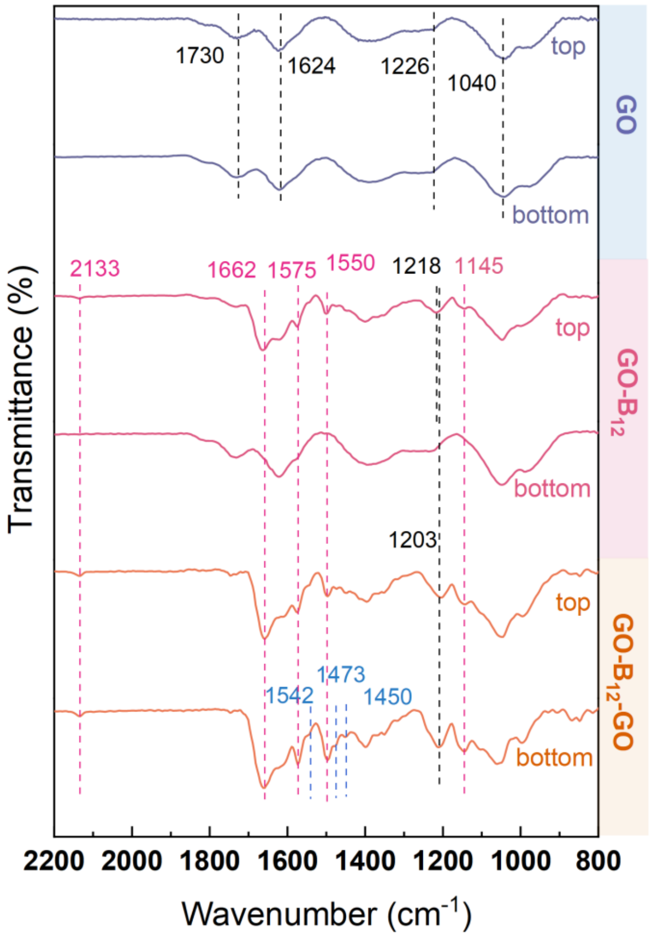

Supplementary Fig. 16 | FTIR spectroscopic measurements conducted on both the top and bottom surfaces of the layered electrodes, including GO, B_{12}, GO-B_{12} and GO-B_{12}-GO.

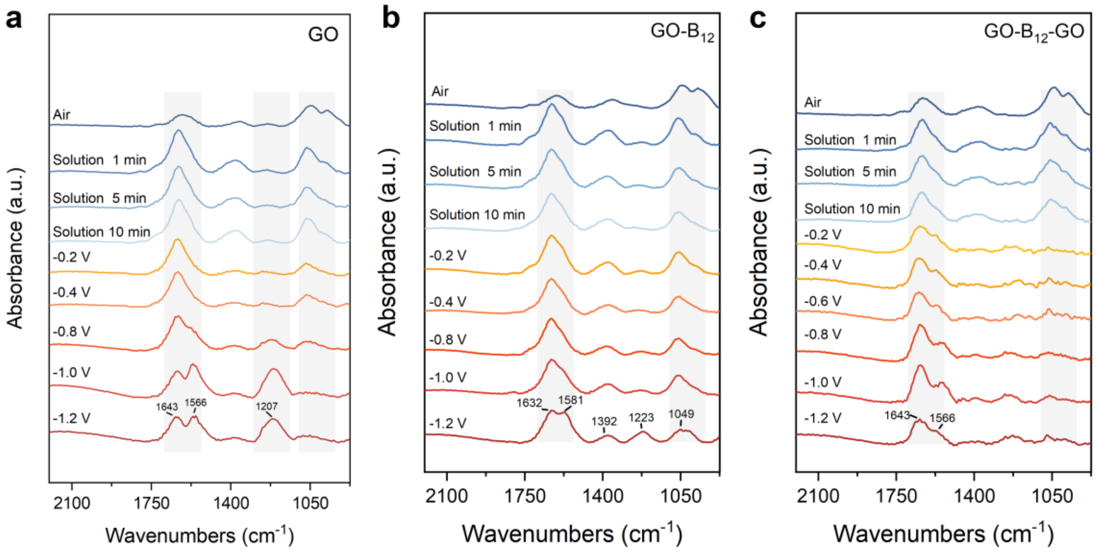

Supplementary Fig. 17 | *In situ* ATR-FTIR spectra of different electrode configurations **a** GO, **b** GO-B_{12} and **c** GO-B_{12}-GO.

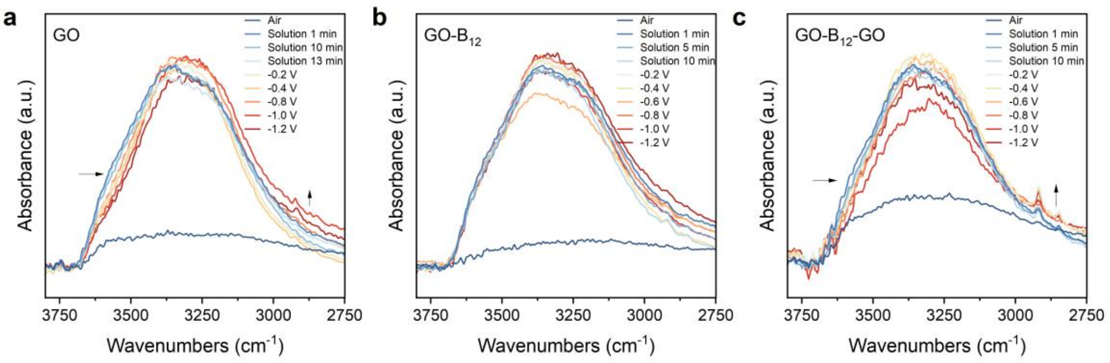

Supplementary Fig. 18 | *In situ* ATR-FTIR spectra of H_2O in different electrode configurations of **a** GO, **b** GO-B_{12} and **c** GO-B_{12}-GO.

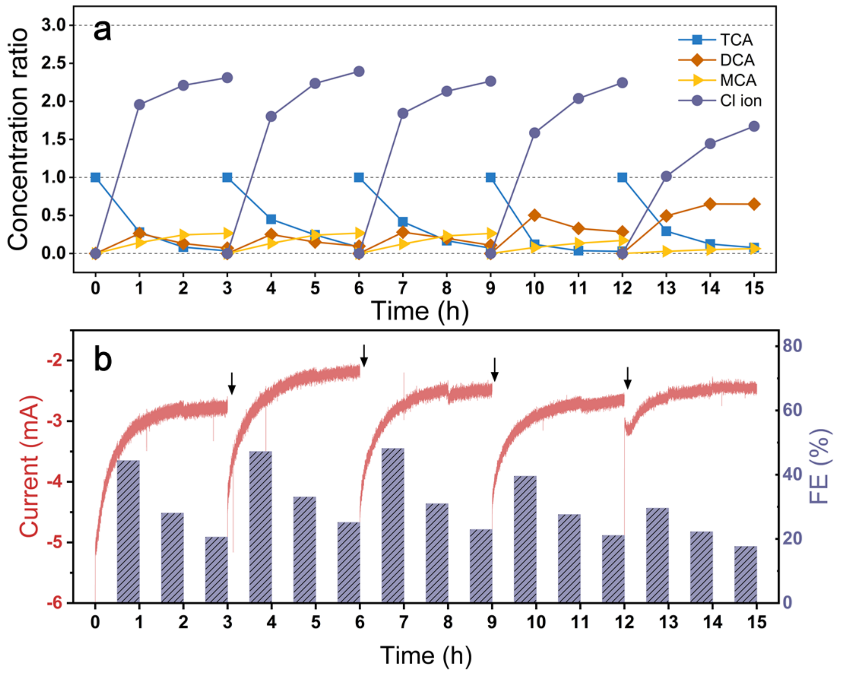

Supplementary Fig. 19 | a The concentration ratios of TCA, DCA, MCA and Cl^-, monitored during the 15-hour electrolysis at the GO-B_{12}-GO electrode. Reaction conditions: an applied potential of -0.9 V *vs*. Ag/AgCl, pH 7.0, and carbon fiber paper as the working electrode. **b** The current-time curves and Faradaic efficiency of the GO-B_{12}-GO electrode, recorded during a 15-hour electrolysis.

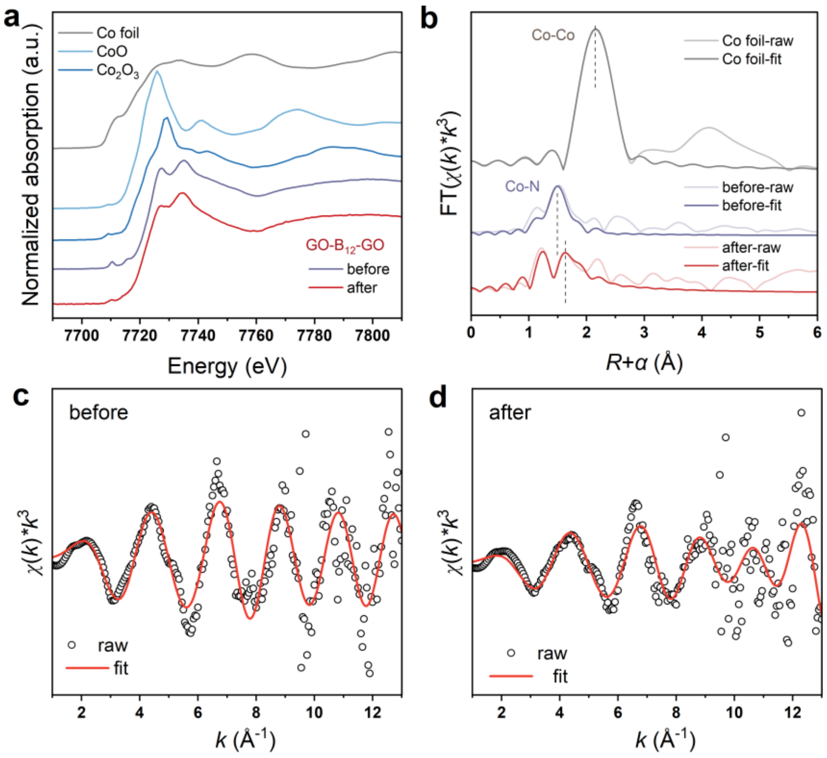

Supplementary Fig. 20 | a Co K-edge XANES experimental spectra of GO-B_{12}-GO electrode before and after electrolysis, CoO, Co_2O_3 and Co foil. **b** Fourier transform of the EXAFS spectrum (k^3-weighted) in R space. **c, d** Fourier transform of EXAFS spectrum in *k* space.

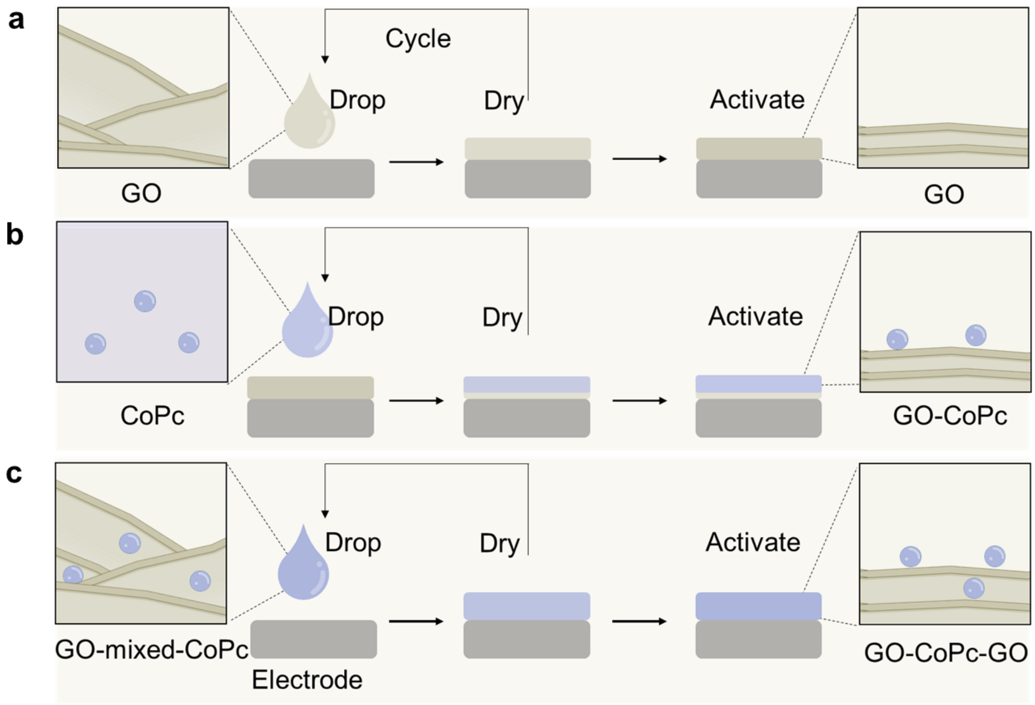

Supplementary Fig. 21 | Schematic illustration of the fabrication of the CoPc-based electrodes, with steps involving drop, dry and electrochemical activation (**a**-**c**).

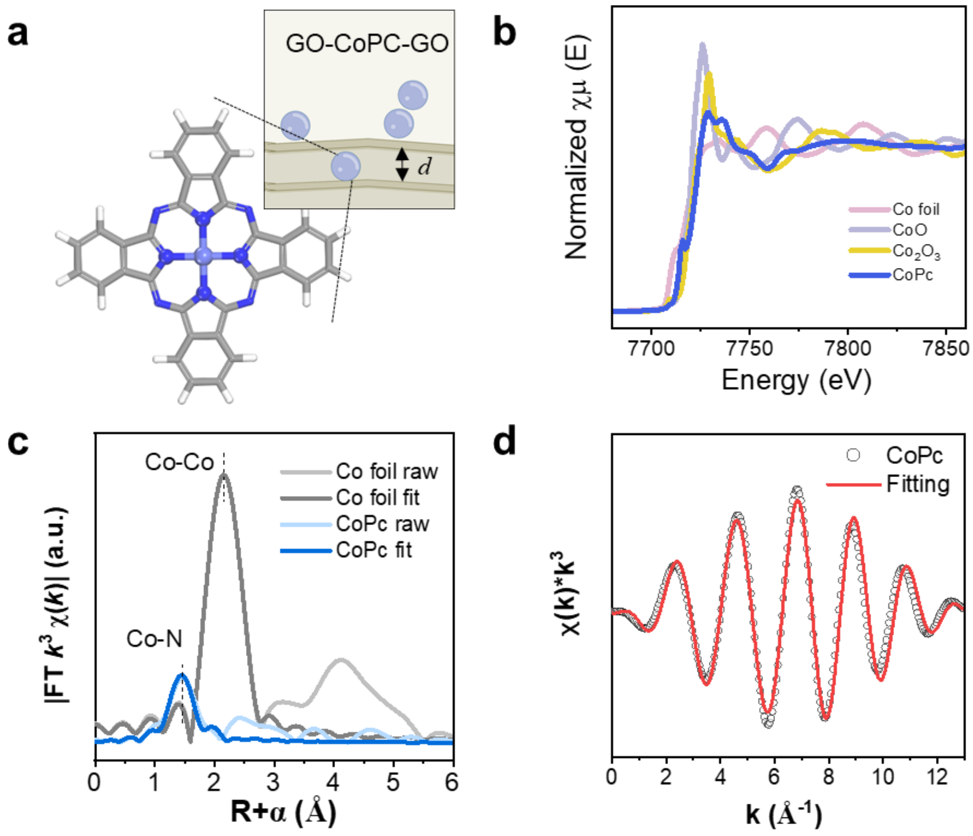

Supplementary Fig. 22 | a Schematic illustration of the GO-CoPc-GO electrode. **b** XANES spectra, **c** Fourier transform (FT) at the Co K-edge of the Co foil and CoPc. **d** The corresponding Co K-edge EXAFS fitting curves of the CoPc.

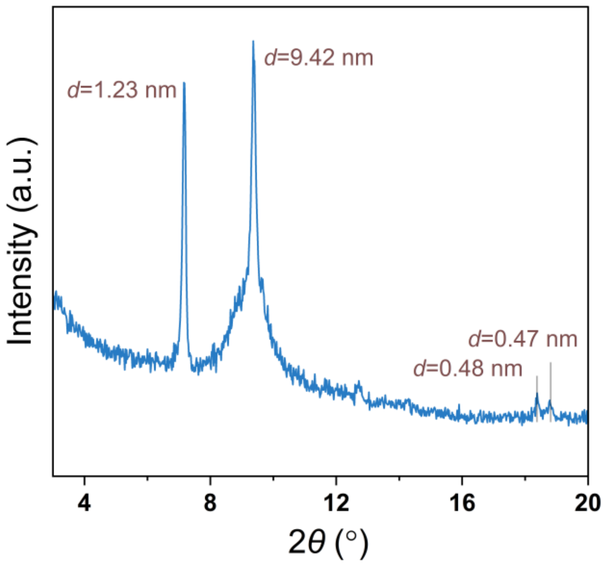

Supplementary Fig. 23 | XRD pattern of GO-CoPc-GO electrode.

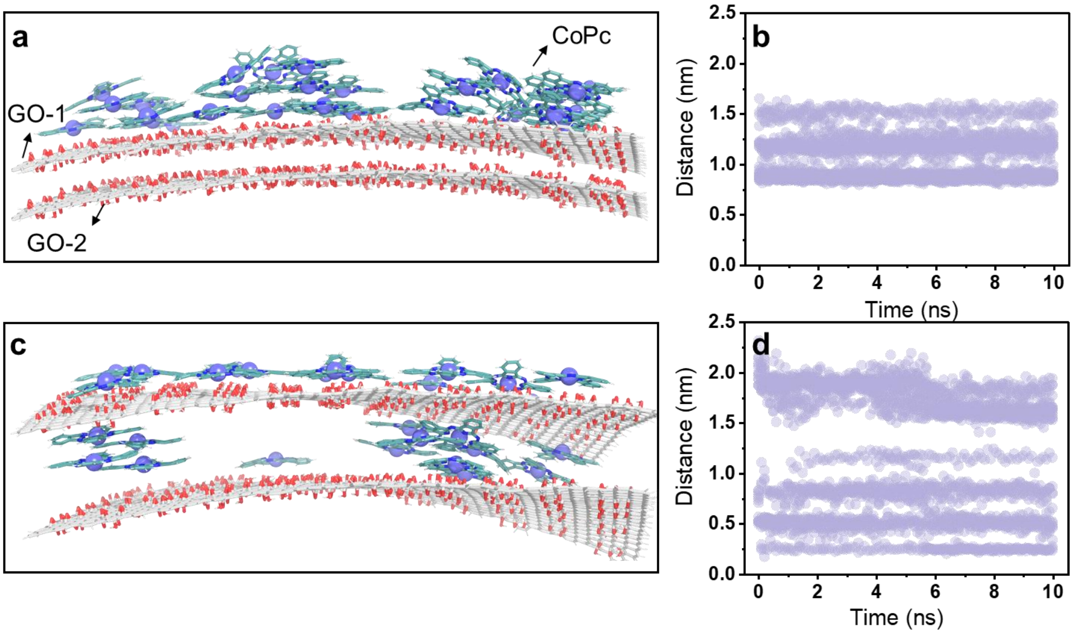

Supplementary Fig. 24 | a, c Snapshots of MD simulations of the GO-CoPc and GO-CoPc-GO electrodes. **b, d** The distance of CoPc-graphene in MD simulations. The GO-2 sheet is selected as the reference graphene. The production run time is 10 ns.

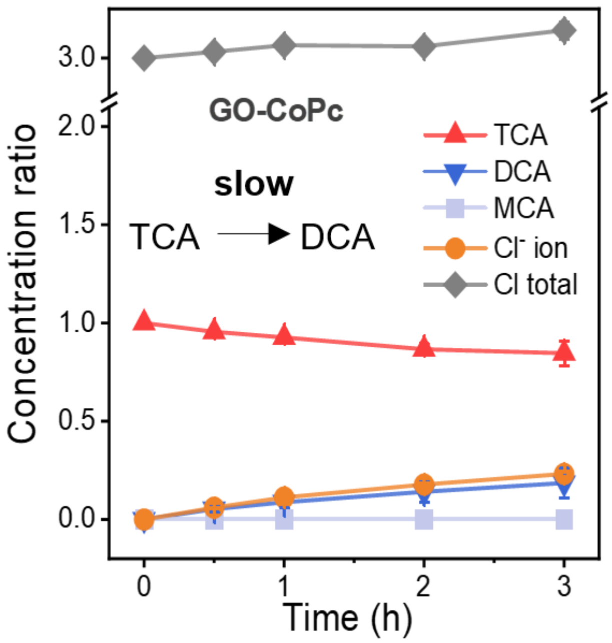

Supplementary Fig. 25 | The mass balance of chlorine element for the electrochemical reduction of TCA at GO-CoPc electrode. The reaction condictions: at –0.29 V *vs*. NHE, pH=7.0. Error bars indicate standard deviations obtained from two independent measurements.

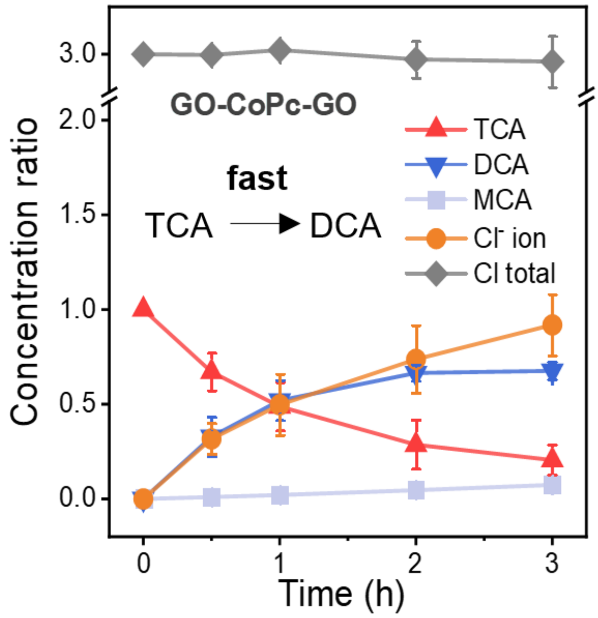

Supplementary Fig. 26 | The mass balance of chlorine element for the electrochemical reduction of TCA at GO-CoPc-GO electrode. The reaction conditions: at −0.29 V *vs.* NHE, pH=7.0. Error bars indicate standard deviations obtained from two independent measurements.

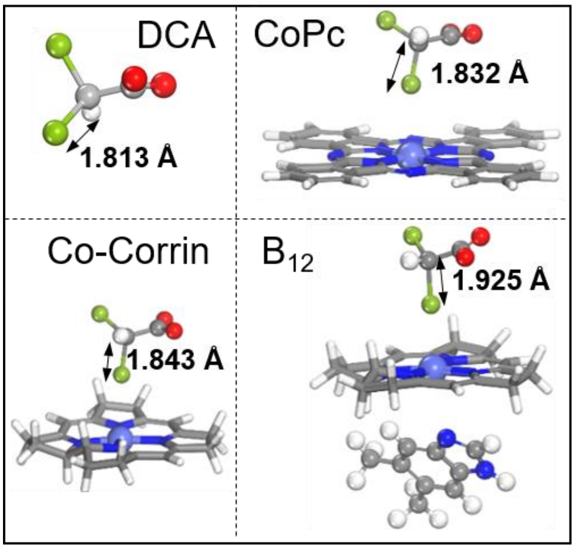

Supplementary Fig. 27 | The optimized structures of DCA alone and the different molecular catalysts of cobalt phthalocyanine (CoPc), Co-centered corrin ring (Co-corrin) and axially 5,6-dimethylbenzimidazole ligated Co-centered corrin (simplified B_{12}). For the model of DCA, light gray, carbon; red, oxygen; green, chlorine.

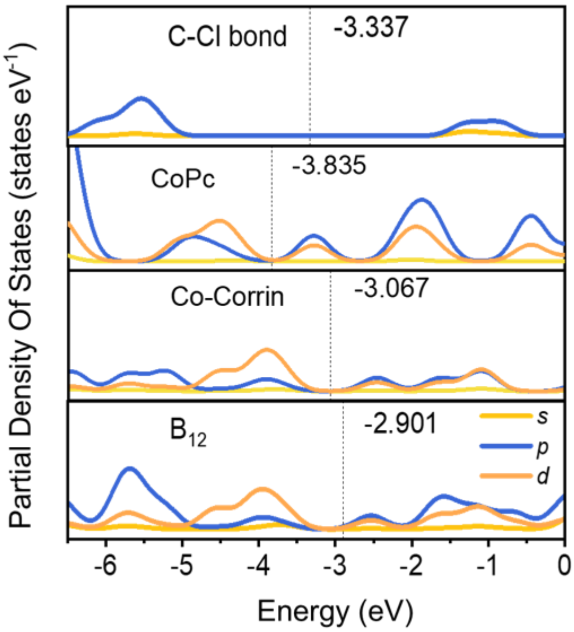

Supplementary Fig. 28 | PDOS for DCA ion, B_{12} and CoPc. In each panel, a dashed vertical line indicates the Fermi level.

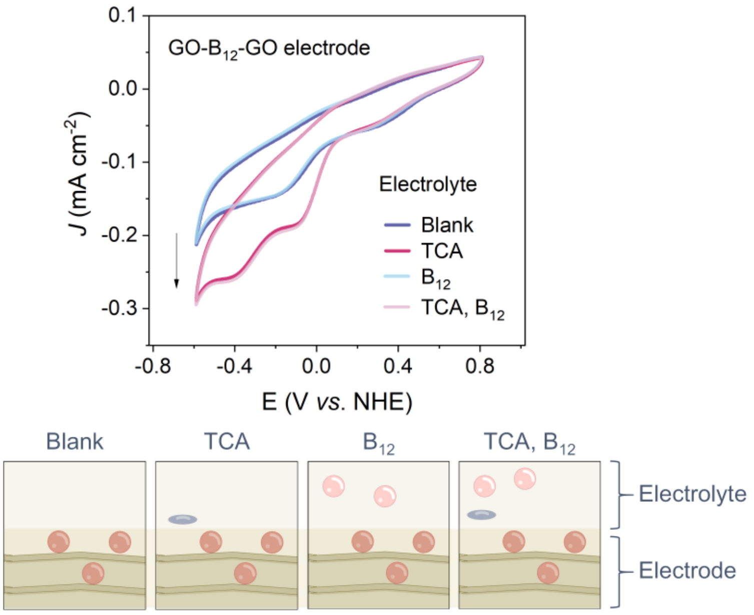

Supplementary Fig. 29 | CV profiles of GO-B_{12}-GO electrode at a scan rate of 200 mV s^{-1}. The components of the electrolyte varied from blank solution to the addition of TCA or B_{12} as well as the co-addition of TCA and B_{12}.

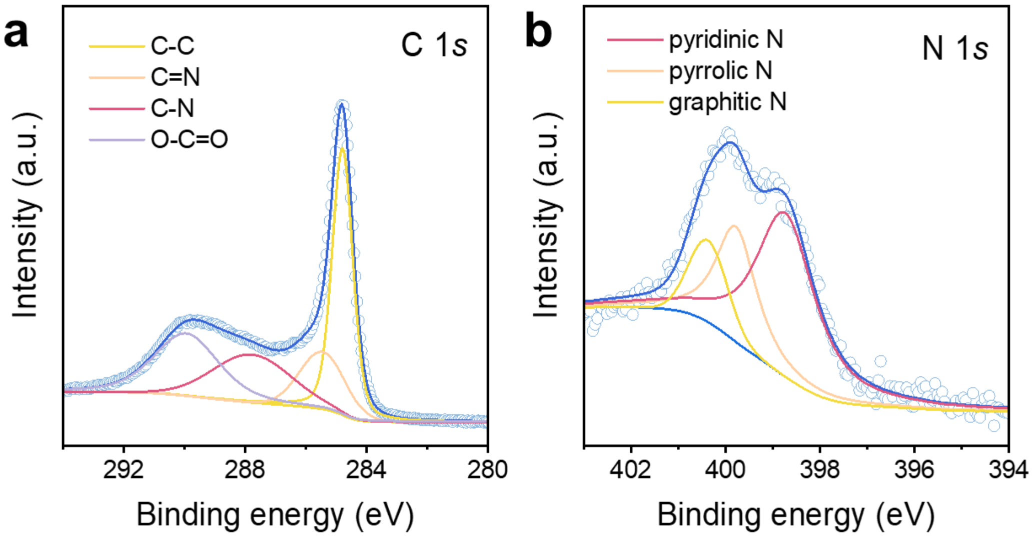

Supplementary Fig. 30 | High-resolution XPS of **a** C 1*s* and **b** N 1*s* for the N-doped GO support.

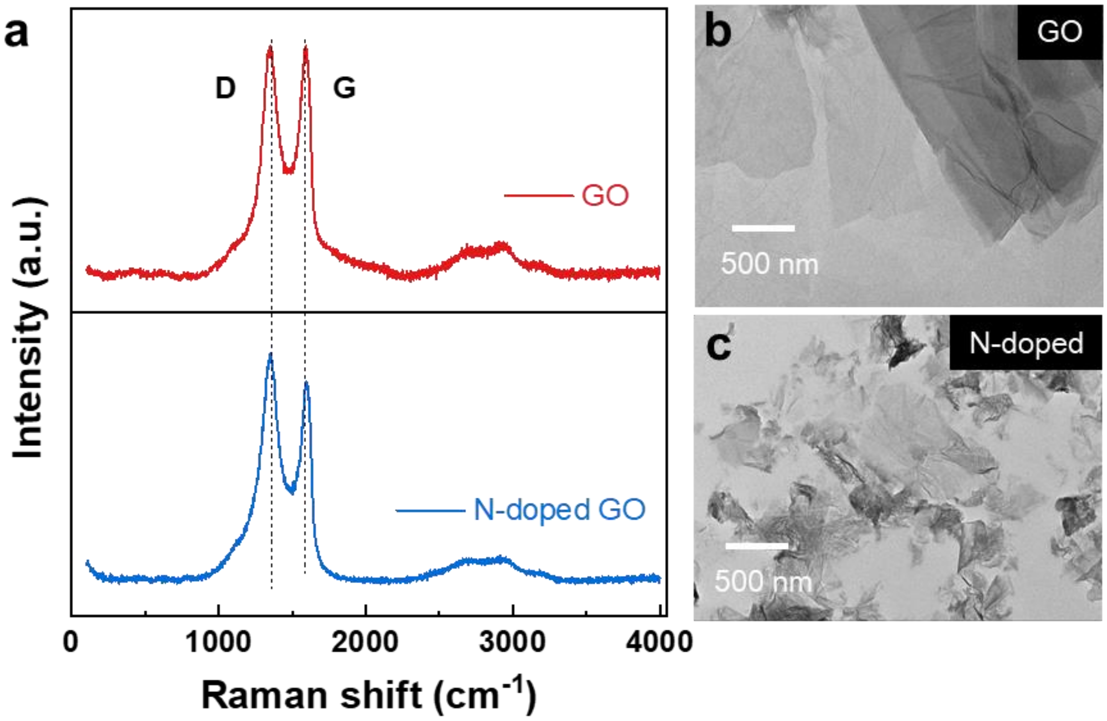

Supplementary Fig. 31 | a Raman spectra and **b, c** TEM images of the graphene oxide (GO) and N-doped graphene (GN).

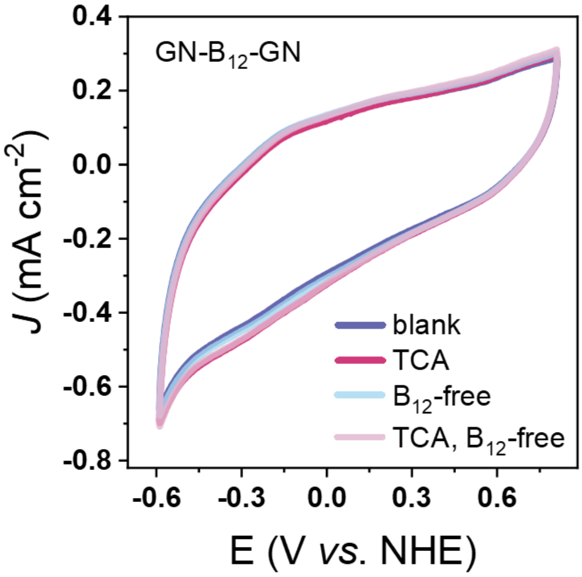

Supplementary Fig. 32 | CV curves of the GN-B_{12}-GN at a scan rate of 200 mV s^{-1}. Glassy carbon electrode was used as the working electrode.

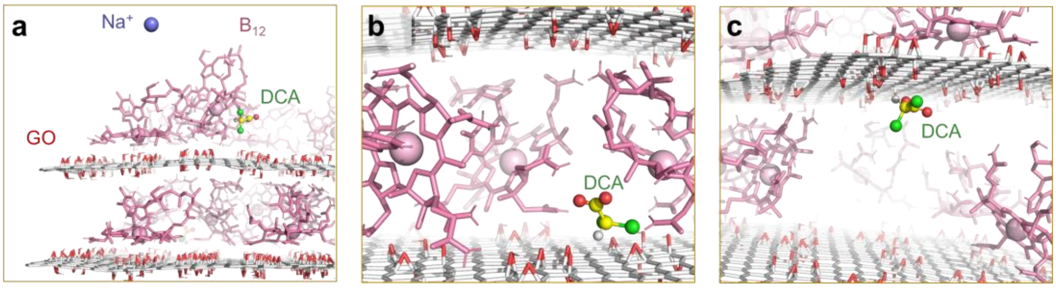

Supplementary Fig. 33 | The snapshots of the MD simulations for the GO-B_{12}-GO and DCA ions in water. **a** The DCA ion adsorbed on the surface. **b, c** The two ions intercalated into the interlayer space. For the model of DCA, yellow, carbon; red, oxygen; green, chlorine. The models of B_{12} are represented in the stick form with cobalt in sphere style (pink). The GO sheets are drawn in the stick representation (light gray, carbon; red, oxygen).

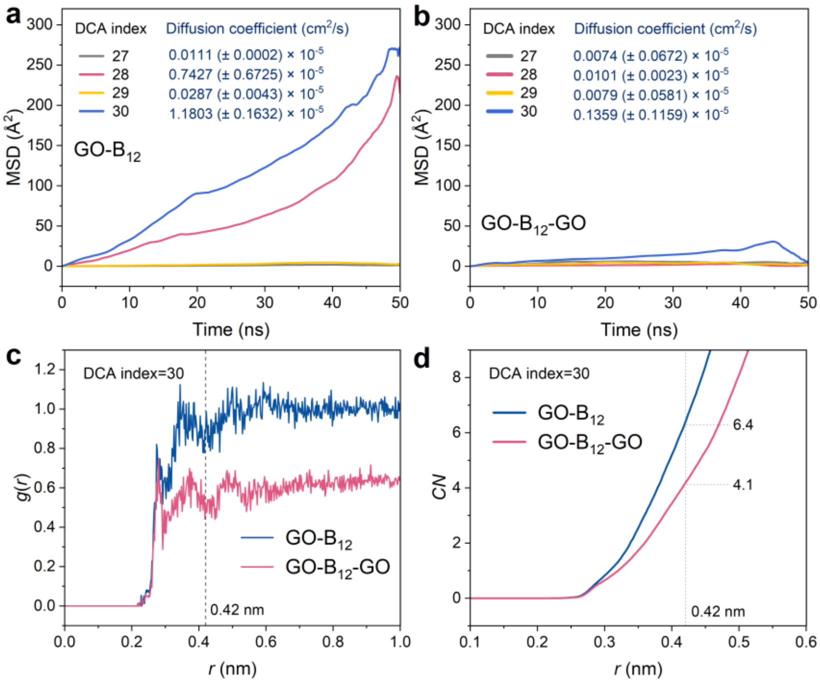

Supplementary Fig. 34 | a, b MSD curves of DCA for analyzing diffusion behavior, **c** radial distribution function of water oxygen around DCA and **d** coordination number of water around DCA at different positions for both GO-B_{12} and GO-B_{12}-GO electrodes.

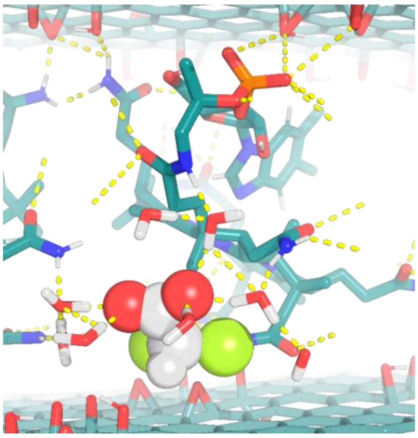

Supplementary Fig. 35 | Plot of the GO-B_{12}-GO electrode with the hydrogen-bond network of the interlayer space (hydrogen bonds: dashed yellow lines). For the model of DCA, light grey, carbon; red, oxygen; green, chlorine. The models of B_{12} and GO are drawn in the stick representation (dark green, carbon).

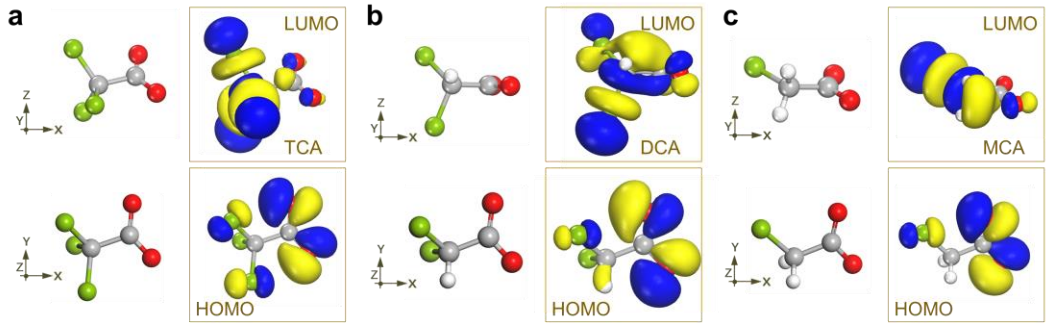

Supplementary Fig. 36 | The LUMO and HOMO of chloroacetate with different numbers of chlorine atoms. **a** TCA, **b** DCA and **c** MCA. For all the three molecules, the LUMO is located at the C–Cl bonds, while the HOMO is located at the C–O bonds. For the model of DCA, light grey, carbon; red, oxygen; green, chlorine. The colors of the orbitals reflect the phases: yellow, negative phase, blue, positive phase (isosurface value = ±0.03 a.u.).

Supplementary Table 1 | EXAFS data fitting results of CoPc and B_{12}.

**Sample**	**Scattering pair**	**CN**	***R*** **(Å)**	σ^2 **(10^{-3}Å^2)**	ΔE_0 **(eV)**	**R factor**
CoPc	Co–N	3.7	1.89	1.9	2.43	0.0018
B_{12}	Co–N	2.1	1.88	3.1	4.91	0.0017

S_0^2 is the amplitude reduction factor (0.8); CN is the coordination number; R is interatomic distance (the bond length between central atoms and surrounding coordination atoms); σ^2 is Debye-Waller factor (a measure of thermal and static disorder in absorber-scatterer distances); ΔE_0 is edge-energy shift (the difference between the zero kinetic energy value of the sample and that of the theoretical model). R factor is used to value the goodness of the fitting. Error bounds that characterize the structural parameters obtained by EXAFS spectroscopy were estimated as CN ± 20%; R ± 1%; σ^2 ± 20%; ΔE_0 ± 20%.

Supplementary References

1 El-Sawy, A. M. et al. Controlling the active sites of sulfur-doped carbon nanotube-graphene nanolobes for highly efficient oxygen evolution and reduction catalysis. *Adv. Energy Mater.* **6**, 1501966 (2016).

2 Schmid, N. et al. Definition and testing of the GROMOS force-field versions 54A7 and 54B7. *Eur. Biophys. J. Biophys. Lett.* **40**, 843-856 (2011).

3 Berendsen, H. J. C., Grigera, J. R. & Straatsma, T. P. The missing term in effective pair potentials. *J. Phys. Chem.* **91**, 6269-6271 (1987).

4 Berendsen, H. J. C., Postma, J. P. M., Vangunsteren, W. F., Dinola, A. & Haak, J. R. Molecular-dynamics with coupling to an external bath. *J. Chem. Phys.* **81**, 3684-3690 (1984).

5 Parrinello, M. & Rahman, A. Polymorphic transitions in single-crystals - a new molecular-dynamics method. *J. Appl. Phys.* **52**, 7182-7190 (1981).

6 Hess, B., Bekker, H., Berendsen, H. J. C. & Fraaije, J. G. E. M. LINCS: A linear constraint solver for molecular simulations. *J. Comput. Chem.* **18**, 1463-1472 (1997).

7 Verlet, L. Computer experiments on classical fluids .I. Thermodynamical properties of Lennard-Jones molecules. *Phys. Rev.* **159**, 98-103 (1967).

8 Darden, T., York, D. & Pedersen, L. Particle mesh ewald - an N.Log(N) method for ewald sums in large systems. *J. Chem. Phys.* **98**, 10089-10092 (1993).

9 Berendsen, H. J. C., Vanderspoel, D. & Vandrunen, R. Gromacs - a message-passing parallel molecular-dynamics implementation. *Comp. Phys. Comm.* **91**, 43-56 (1995).

10 Humphrey, W., Dalke, A. & Schulten, K. VMD: Visual molecular dynamics. *J. Mol. Graph. Model.* **14**, 33-38 (1996).

11 Schrodinger, LLC. The PyMOL molecular graphics system, Version 1.8. (2015).

12 Schrodinger, LLC. The AxPyMOL molecular graphics plugin for microsoft powerpoint, Version 1.8. (2015).

13 Delley, B. From molecules to solids with the $DMol^3$ approach. *Journal of Chemical Physics* **113**, 7756-7764 (2000).

14 Perdew, J. P., Burke, K. & Ernzerhof, M. Comment on "Generalized gradient approximation made simple" - Reply. *Phys. Rev. Lett.* **80**, 891-891 (1998).

15 Koelling, D. D. & Harmon, B. N. A technique for relativistic spin-polarised calculations. *J. Phys. C: Solid State Phys.* **10**, 3107-3114 (1977).

16 Tkatchenko, A. & Scheffler, M. Accurate molecular van der Waals interactions from ground-state electron density and free-atom reference data. *Phys. Rev. Lett.* **102**, 073005 (2009).

17 Klamt, A. & Schüürmann, G. COSMO: a new approach to dielectric screening in solvents with explicit expressions for the screening energy and its gradient. *J. Chem. Soc., Perkin Trans. 2*, 799-805 (1993).

18 Voiry, D. et al. High-quality graphene via microwave reduction of solution-exfoliated graphene oxide. *Science* **353**, 1413-1416 (2016).
19 Chang, S. T. et al. Vitalizing fuel cells with vitamins: pyrolyzed vitamin B_{12} as a non-precious catalyst for enhanced oxygen reduction reaction of polymer electrolyte fuel cells. *Energ Environ Sci* **5**, 5305-5314 (2012).
20 Kumar, P. V. et al. Scalable enhancement of graphene oxide properties by thermally driven phase transformation. *Nat. Chem.* **6**, 151-158 (2014).
21 Pei, S. F., Wei, Q. W., Huang, K., Cheng, H. M. & Ren, W. C. Green synthesis of graphene oxide by seconds timescale water electrolytic oxidation. *Nat. Commun.* **9**, 145 (2018).
22 Acik, M. et al. The role of intercalated water in multilayered graphene oxide. *ACS Nano* **4**, 5861-5868 (2010).
23 Bregante, D. T. et al. The shape of water in zeolites and its impact on epoxidation catalysis. *Nat. Catal.* **4**, 797-808 (2021).
