# Supplementary material for: Mimicking reductive dehalogenases for efficient electrocatalytic water dechlorination
Source: Nat Commun. 2023 Aug 23;14:5134. doi: 10.1038/s41467-023-40906-6 (PMC10447495; doi:10.1038/s41467-023-40906-6)
Supplement: Supplementary file 3 — Description of Additional Supplementary Files [file 41467_2023_40906_MOESM3_ESM.pdf]

Description of Additional Supplementary Files

File Name: Supplementary Movie 1
Description: The trajectory of GO-B_{12}-GO vdW heterostructure with DCA ions. The GO layers are in yellow and the B_{12} in pink. The Na^+ ions and water molecules are hidden for a better visualization. DCA ions could be intercalated into the inner space between the GO layers after equilibrium.

File Name: Supplementary Movie 2
Description: The trajectory of GO-B_{12} vdW heterostructure with DCA ions. With limited basal spacing, the DCA ions are excluded from the interlayer space.
